# Term Infant with Gallbladder Stone: a Case Report

**Published:** 2014-06

**Authors:** Ali Ulas Tugcu, Aslıhan Abbasoğlu, Filiz Yanık, Ayşe Ecevit, Aylin Tarcan

**Affiliations:** 1 Department of Pediatrics, Division of Neonatology; 2 Department of Obstetrics and Gynecology, Divisions of Perinatology, Faculty of Medicine, Baskent University Ankara Hospiatal, Ankara, Turkey

**Keywords:** Gallbladder Stone; Hyperbilirubinemia; Infant

**To the Editor**

Fetal gallbladder stone is a rare phenomenon, which is observed by chance during third trimester ultrasonography and does not cause significant clinical symptoms. Even though the etiology of stones found in the fetal period are yet unknown; apnea of prematurity, sepsis, parenteral nutrition, motile diseases, blood group incompatibilities, metabolic diseases and dehydration of newborns are among the causes of formation of gallstones in pediatric age groups.

 We present a case where the mother had used high dose flaxseed oil capsules, which are widely used as sources of omega-3 and omega-6 during pregnancy. Antenatal ultrasonography determined an echogenic focus beneath the liver position of the fetus. This was found to be gallbladder mud and stone with abdominal ultrasonography on the 2nd day of life. 

**Fig. 1:**
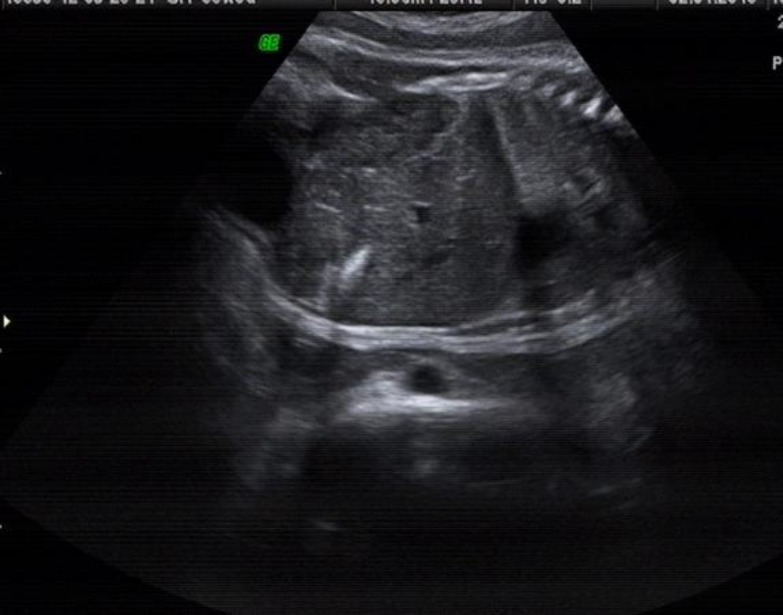
Hyperechogenic foci beneath the liver

 This term (38 weeks and 5 days, 3300 g), appropriate for gestational age, male infant was born to a 33-year-old mother who used linseed (flaxseed) oil capsules 2-3 times (2000-3000 mg) a day during pregnancy for a period of 7 months. During antenatal period, when the fetus had gestanional age of 36 weeks; a hyperechogenic focus was showed beneath the liver position of the fetus. After birth, patient was evaluated and abdominal ultrasonography revealed “gallbladder mud and stone”(Fig .1). Biochemical parameters including liver, function test, total cholesterol and triglycerides were in normal range but total bilirubin and direct bilirubin were 6 mg/dl and 0.6 mg/dl, respectively.

In full blood count, Hemoglobin was 16.7 g/dl, reticulocytes 5.81%, platelet count 245000/mm^3^ and white blodd cells 14400/mm^3^. C-reactive protein value and direct Coombs test were negative. A peripheral blood smear examination did not include hemolytic findings. There had been no pyruvate kinase and pyrimidine 5’ nucleotides deficiency of patient whose glucose 6 phosphate dehydrogenase level was 12 U/g HB. Tandem mass spectrometry, urine organic acid and urine blood amino acid tests were normal.

 Phototherapy was administered for three days due to indirect hyperbilirubinemia. After discharge on the 7^th^ postnatal day, patient was found well-fed and had gained weight. During follow-up, abdominal ultrasonography revealed a normal gallbladder without gallstones at 1 month of age. 

 Fetal gallbladder stone was defined for the first time by Beretski and Lankin in 1983^[^^1^^]^. Its prevalence is not known precisely. Its occurrence rate in the literature is low. Agnifili et al reported fetal gallbladder stone incidence as 0.39%^[^^2^^]^. The widest series was reported by Brown and colleagues, who detected echogenic foci in 25 fetus’ gallbladder^[^^3^^]^.

 None of the many hypotheses suggested can give a possible explanation for fetal gallbladder stone formation. Fanaroff and colleagues represent the idea that bilirubin, a breakdown product of hemoglobin, causes indirect bilirubin level increase by accessing the fetus through the placenta and that this causes fetal gallbladder stone formation^[^^4^^]^.

 It has been asserted that smoking during pregnancy, hematologic diseases, blood incompatibilities between mother and fetus, and structural anomalies like choledochal cysts may cause formation of fetal gallbladder stones^[^^5^^]^.

 On the other hand, Brown and colleagues proposed that high estrogen levels increase cholesterol secretion and decrease bile acid production and that this mechanism might cause pigment stones in gallbladder ^[^^3^^]^. 

 Flaxseed (*Linum usitatissimum L., Linaceae) *is a vegetable product, which contains high quantity of alpha linolenic acid that can be converted into omega-3. Phytoestrogens are polyphenolic nonsteroidal vegetable origin compounds which structurally and functionally resemble β-estradiol found in mammals^[^^6^^]^. Phytoestrogens have both agonistic and antagonistic effects on estrogen receptors. These behave like endogen estrogens as agonists and trigger estrogenic effects. Flaxseed contains high level phytoestrogen which is in lignan structure (8 mg/g secoisolariciresinol dry weight) which mimics the structure of 17 β-estradiol and synthetic estrogen “diethyl-stilbestrol”. Breakdown products of lignans also have estrogenic activities^[^^6^^]^.

 This is more likely due to high omega-3 content, flaxseed is generally preferred during pregnancy. But there is controversy in consuming flaxseed during pregnancy and lactation. It has been proved that flaxseed would pass from mother to baby during pregnancy and from breast milk following birth. However it is believed that its phytoestrogen content may increase rates of breast, prostate and endometrium cancer, as well as cause truncal obesity, hypertension, anabolic activity increase and stone formation in gallbladder due to its estrogenic activity^[^^7^^]^.

 For the patient we mentioned, high consumption of flaxseed may have caused stone formation in the gallbladder because of its phytogenic lignan structure which increases estrogenic activity.
